# Ocular Vaccinia Infection in Laboratory Worker, Philadelphia, 2004

**DOI:** 10.3201/eid1201.051126

**Published:** 2006-01

**Authors:** Felicia M.T. Lewis, Esther Chernak, Erinn Goldman, Yu Li, Kevin Karem, Inger K. Damon, Richard Henkel, E. Claire Newbern, Patrina Ross, Caroline C. Johnson

**Affiliations:** * Philadelphia Department of Public Health, Philadelphia, Pennsylvania, USA;; † Centers for Disease Control and Prevention, Atlanta, Georgia, USA

**Keywords:** Poxviridae infections, vaccinia virus, laboratory-acquired infection, vaccinia immune globulin, biosafety practices, dispatch

## Abstract

We report a case of ocular vaccinia infection in an unvaccinated laboratory worker. The patient was infected by a unique strain used in an experiment performed partly outside a biosafety cabinet. Vaccination should continue to be recommended, but laboratories with unvaccinated workers should also implement more stringent biosafety practices.

Vaccinia virus, the orthopoxvirus used in smallpox vaccine, is increasingly used in research laboratories, both to investigate orthopoxvirus biology and as a tool in molecular biology and immunology (*1**–**4*). Vaccinia can cause mild-to-moderate infection in healthy hosts and can be transmitted to their contacts (*3**,**5**–**8*). Although routine smallpox vaccination has been discontinued in the United States since 1971, vaccination is still recommended for healthcare and laboratory workers who handle nonattenuated orthopoxviruses (*6*). We document ocular vaccinia infection in an unvaccinated laboratory worker and describe the associated laboratory and epidemiologic investigation.

## Case Report

An immunology graduate student born after the discontinuation of routine smallpox vaccination was working with multiple strains of vaccinia as part of her thesis research. She had voluntarily declined vaccination before beginning laboratory work with vaccinia. One morning in October 2004, she noticed the onset of itching, tearing, palpebral swelling, and conjunctival injection in her left eye. Viral conjunctivitis was diagnosed by her student health services, and over-the-counter tetrahydrozoline hydrochloride eye drops were prescribed. During the next 4 days, the eye became swollen, red, and painful; malaise, fatigue, and subjective fever also appeared. On day 5 the patient went to a private ophthalmologist, who referred her to a specialty eye hospital.

**Figure 1:**
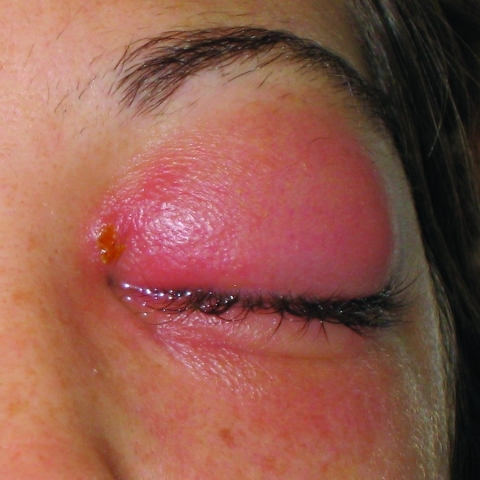
Patient's left eye after admission to hospital. The primary pox lesion is located at the inner canthus. Photographer: E. Claire Newbern.

Physical examination at the eye hospital demonstrated a painful left eye with 3+ chemosis in the eyelids and conjunctiva and symblepharon at the lower pole of the eye. A 0.5-cm vesicle was noted above the left canthus (Figure 1). Left ocular range of motion, including palpebral motion, was severely limited. Keratitis was not evident. Routine laboratory values were normal. Computed tomographic scan of the orbits indicated left preseptal cellulitis without evidence of orbital cellulitis. The diagnosis of vaccinia infection was not suspected until examination at the eye hospital, when the student first mentioned her work with vaccinia. Contact precautions were then initiated, and a scraping of the vesicle above the canthus was sent to the Pennsylvania Bureau of Laboratories for vaccinia testing. The patient was started on trifluridine and bacitracin ointments, broad-spectrum systemic antimicrobial agents, and pain medication; she was admitted to the hospital.

**Figure 2:**
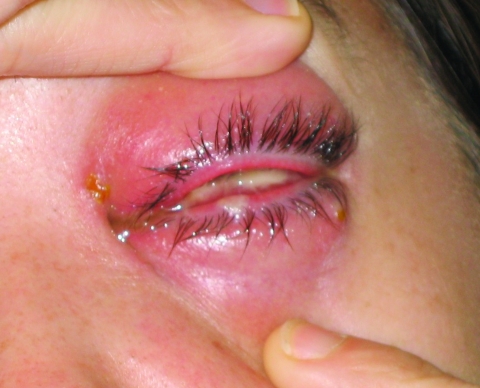
Satellite lesion on lower conjunctiva. Photographer: E. Claire Newbern.

During the next 48 hours, additional vesicles appeared on the lower conjunctiva (Figure 2), and periorbital swelling increased. Polymerase chain reaction (PCR) testing at the Pennsylvania Bureau of Laboratories showed evidence of vaccinia; results were confirmed at the Centers for Disease Control and Prevention (CDC). At this time, vaccinia immune globulin (VIG), 6,000 U/kg IV, was administered. Less than 24 hours after VIG administration, the patient's pain and swelling were substantially decreased. The patient continued to improve over the next 2 days and was discharged to her home on day 9. No long-term sequelae occurred, although recovery took several weeks.

A public health investigation of the patient's home and work contacts and the research laboratory was initiated. Because cutaneous lesions from vaccinia typically appear 3–5 days after inoculation (*5*), investigators postulated that the patient contracted her infection within the 7 days before symptom onset. The patient was considered infectious from the beginning of this period until hospitalization (11 days). During this time, she had 3 household contacts and 11 work contacts. Household contacts were monitored for signs of illness for 1 week. All contacts were interviewed with a standard questionnaire to ascertain the extent of their contact with the patient, vaccination status, and information about laboratory work practices, if applicable. Acute- and convalescent-phase paired serum samples were drawn at 1 and 6 weeks after exposure from all work contacts, as well as from the patient's closest home contact. Convalescent-phase serum was drawn from the patient at week 6. Paired serum samples from the patient's consenting contacts were examined for evidence of orthopoxvirus-reactive antibody by using techniques described elsewhere (*9*). Serologic results are summarized in the Table.

**Table:** Vaccination status and serologic evidence of vaccinia immunity of patient and contacts

Patient and contact	Prior vaccination	Date of last vaccination	Anti-orthopoxvirus IgG present* (acute-phase serum, 10/04; convalescent-phase serum, 12/04)	Anti-orthopoxvirus IgM present† (acute-phase serum 10/04; convalescent-phase serum, 12/04)
Patient	No	–	–/Yes	–/Yes
Home	No	–	No/no	No/no
Worker 1	Yes, 5×‡	1994	Yes/yes	No/no
Worker 2§	Yes, as child	12/01/04	Yes/yes	No/no
Worker 3§	Yes, as child	12/01/04	Yes/yes	No/no
Worker 4§	Yes, as child	12/01/04	No/no	No/no
Worker 5	No	–	No/no	No/no
Worker 6	No	–	No/no	No/no
Worker 7	No	–	No/no	No/no
Worker 8	No	–	No/no	No/no
Worker 9	No	–	No/no	No/no
Worker 10	No	–	No/no	No/no
Worker 11	No	–	No/no	No/no

*Immunoglobulin G (IgG) optical density cutoff value (COV) = 0.214408; –, not performed. †IgM optical density COV = 0.015763. ‡The last vaccination was ≈10 years before this incident. §Three laboratory workers who also manipulated vaccinia were vaccinated 1 week before the convalescent-phase blood sample was drawn (workers 2, 3, and 4). All 3 had been vaccinated as children; workers 2 and 3 had orthopoxvirus-reactive IgG levels present above the COV. Worker 4 did not have IgG levels above the COV either before or after her recent vaccination.

A laboratory inspection, which included a review of experiments performed by the patient during the week before symptom onset, was conducted. Although laboratory staff generally followed established biosafety precautions (*10*), review of laboratory practices showed several opportunities for virus exposure. Staff infrequently wore eye protection while performing experiments with vaccinia. Laboratory coat sleeves were not elasticized and did not always cover the wrist. Waste pipettes were not disinfected before removal from the biosafety cabinet. Instances occurred in which samples with low titers of live virus were removed from the biosafety cabinet, transported to other parts of the facility, and manipulated. In addition, laboratory staff routinely vortexed tubes containing live virus outside of the biosafety cabinet. Most important, no laboratory workers had been vaccinated in the past 10 years, as recommended by CDC (*6**,**10*).

To identify the specific infecting strain of vaccinia, the virus isolated from the patient's canthus lesion was sequenced. Briefly, a 3.7-kbp amplicon was generated and sequenced from the thymidine kinase region of the viral genome by using the following primers: TKj2r forward 5-ACGTG ATGGA TATAT TAAAG TCGAA and TKj2r reverse 5-GTTTA TCTAA CGACA CAACA TCCA. Amplification was performed with the Expand Long Template PCR kit (Roche Molecular Biologicals, Indianapolis, IN, USA) and a Cetus Model 9700 thermocycler (Perkin-Elmer Life and Analytical Sciences, Boston, MA, USA) at 92°C × 2 min, followed by 30 cycles of 92°C × 10 s, 55°C × 30 s, and 68°C × 3 min. Purified, amplified DNA was sequenced with a CEQ 8000 Genetic Analysis System (Beckman-Coulter, Fullerton, CA, USA). Sequences were assembled using SeqMan software (DNASTAR, Inc., Madison, WI, USA).

Sequencing showed that the infecting virus was a unique form of recombinant Western Reserve vaccinia constructed in the research laboratory and routinely used by the patient; it had been last used as a control strain during a multiday experiment performed in the 5 days before the patient's symptoms began. At one point in this experiment, a 96-well plate containing small amounts of live vaccinia–infected mammalian cells was removed from the biosafety cabinet and hand-carried to another room, where the lid of the plate was removed, and the cells were examined for fluorescence. The student did not wear eye protection during this phase of the experiment; whether she wore gloves is unclear.

## Conclusions

The investigation of the laboratory and examination of clinical specimens from the patient and contacts enabled investigators to pinpoint the source of infection to a single experiment. During the period when the patient could have become infected, she was the only laboratory member to use the culprit vaccinia strain, and she used it only while performing this particular experiment. Lack of seroconversion among the other staff argues against widespread environmental viral contamination in the laboratory. During the time when she could have become infected, the student had also worked with a different strain of vaccinia in titers as high as 1×10^10^ PFU/mL. However, all of the work with virus at this titer occurred in the biosafety cabinet.

Although the exact mechanism of infection could not be determined, the location of the principal lesion at the inner canthus suggests either inadvertent inoculation from hand to eye or inoculation through aerosolization of virus (*5*). Regardless, both mechanisms indicate that existing biosafety precautions in the laboratory were likely insufficient. Biosafety level 2 (BSL-2) precautions are recommended for laboratories and persons who manipulate nonattenuated strains of vaccinia virus (*10*). This recommendation assumes a priori that all such persons will be adequately vaccinated against the virus. However, this report and others of laboratory-acquired vaccinia infections demonstrate that vaccination is being waived in certain institutions (*1**–**3**,**11*). No current recommendations exist in the United States for the level of precautions to be used by unvaccinated personnel. We believe that vaccination would probably have prevented or attenuated this patient's infection and that it should continue to be recommended for laboratory workers who handle vaccinia. However, given that vaccination has risks of its own that might reduce its use (including a rate of ocular complications of 10–20/1 million immunizations) (*5**,**6**,**12**,**13*), biosafety recommendations for unvaccinated personnel should be specifically addressed.

Chiefly intended to protect against agents with potential for respiratory transmission, BSL-3 precautions emphasize protection from exposure to potentially infectious aerosols (*10*). CDC has previously recommended increased biosafety precautions for laboratories with unvaccinated personnel who manipulate monkeypox virus (*14*). Implementing certain BSL-3 precautions in this case, e.g., performing all manipulations of virus in the biosafety cabinet or other enclosed equipment, frequent glove changing accompanied by handwashing, and always wearing goggles or face shields when working with virus outside of a primary containment device, would have minimized the potential for human error and might have prevented this infection. Use of eye protection should be particularly stressed, as serious eye infections can occur even in previously vaccinated persons (*15*). No systematic monitoring of vaccinia infection in laboratory workers currently exists, so the full extent of the problem is unknown. Further investigation of laboratory practices involving vaccinia is warranted. At the present time, vaccination is the best way to prevent or mitigate accidental infection (*4*) and should continue to be recommended for laboratory workers handling nonattenuated strains of vaccinia. If vaccination is impossible, workers should implement more stringent biosafety practices, such as consistently using goggles and performing all manipulations of virus in the biosafety cabinet.
